# Beyond the weight loss: revisiting small bowel obstruction as an underestimated risk of intragastric balloons

**DOI:** 10.1093/jscr/rjaf998

**Published:** 2025-12-18

**Authors:** Santiago Muñoz-Palomeque, Diego Molina-Mosquera, Felipe Pacheco-Barzallo

**Affiliations:** Department of Surgery, Hospital Metropolitano, Quito, 170508, Ecuador; Faculty of Medical, Health and Life Sciences, Universidad Internacional del Ecuador, Quito, 170411, Ecuador; Department of Surgery, Division of General Surgery, Hospital Metropolitano, Quito, 170508, Ecuador; Clinica Pasteur, Quito, Ecuador; Department of Surgery, Division of General Surgery, Hospital Metropolitano, Quito, 170508, Ecuador; Clinica Pasteur, Quito, Ecuador

**Keywords:** intragastric balloon, small bowel obstruction, migration, bariatric therapy, enterotomy

## Abstract

Intragastric balloons (IGB) are a minimally invasive, reversible method for weight reduction in obese patients who fail conservative therapy but do not qualify for bariatric surgery. Despite their safety profile, rare yet severe complications can occur, such as small bowel obstruction (SBO) due to balloon migration. We present a case of a 37-year-old woman who developed SBO one year after gastric balloon placement, following successful weight loss. Imaging revealed a migrated balloon obstructing the jejunum, which required surgical extraction via enterotomy. This report compares our case with previously documented events, highlighting the clinical presentation, diagnostic challenges, and therapeutic decision-making. Although reported rates of obstruction remain below 0.2%, migration may occur months after the expected balloon excretion period. Persistent vigilance is required even long after IGB therapy. A stepwise diagnostic and management algorithm is proposed to guide clinicians facing this rare but potentially life-threatening complication.

## Introduction

Obesity remains a major public health issue worldwide. Bariatric surgery is the most effective and durable intervention, but eligibility criteria—body mass index (BMI) ≥40 kg/m^2^ or ≥ 35 kg/m^2^ with comorbidities—exclude many patients who fail medical management consisting in lifestyle and pharmacologic interventions. For these individuals, intragastric balloon (IGB) therapy offers a minimally invasive, temporary option to induce weight loss by reducing gastric capacity and delaying gastric emptying [1].

IGBs induce early satiety by mechanically reducing gastric capacity and delaying gastric emptying [1]. Devices such as the Elipse™ swallowable intragastric balloon (EIGB) represents a significant advance, as it is designed for non-endoscopic placement and spontaneous excretion after ~16 weeks. A systematic review of 2013 patients by Vantanasiri *et al*. [2] found EIGB to be safe and effective, with a small-bowel obstruction (SBO) rate of only 0.13% (n = 3). Similarly, Ienca *et al*. [3] reported on 1770 consecutive patients and demonstrated an excellent safety profile with 14.2% total body weight loss (TBWL) and only three cases of SBO requiring surgical removal. Alsabah *et al*. [4] corroborated these findings, noting one SBO among 135 patients.

Nonetheless, rare yet serious adverse events—including migration and intestinal obstruction—continue to challenge the perceived safety of these devices. We describe a case of complete small bowel obstruction due to a migrated gastric balloon one year after placement, emphasizing diagnostic reasoning, surgical management, and lessons learned from literature comparison resumed in a practical guideline.

## Case report

A 37-year-old woman with a history of gastric balloon placement one year prior for grade I obesity (initial BMI = 30.5 kg/m^2^) presented with five days of bilious vomiting and abdominal pain, worsened in the preceding 48 hours, along with inability to pass flatus. Her BMI at presentation was 24.09 kg/m^2^, consistent with sustained post-balloon weight reduction.

**Figure 1:**
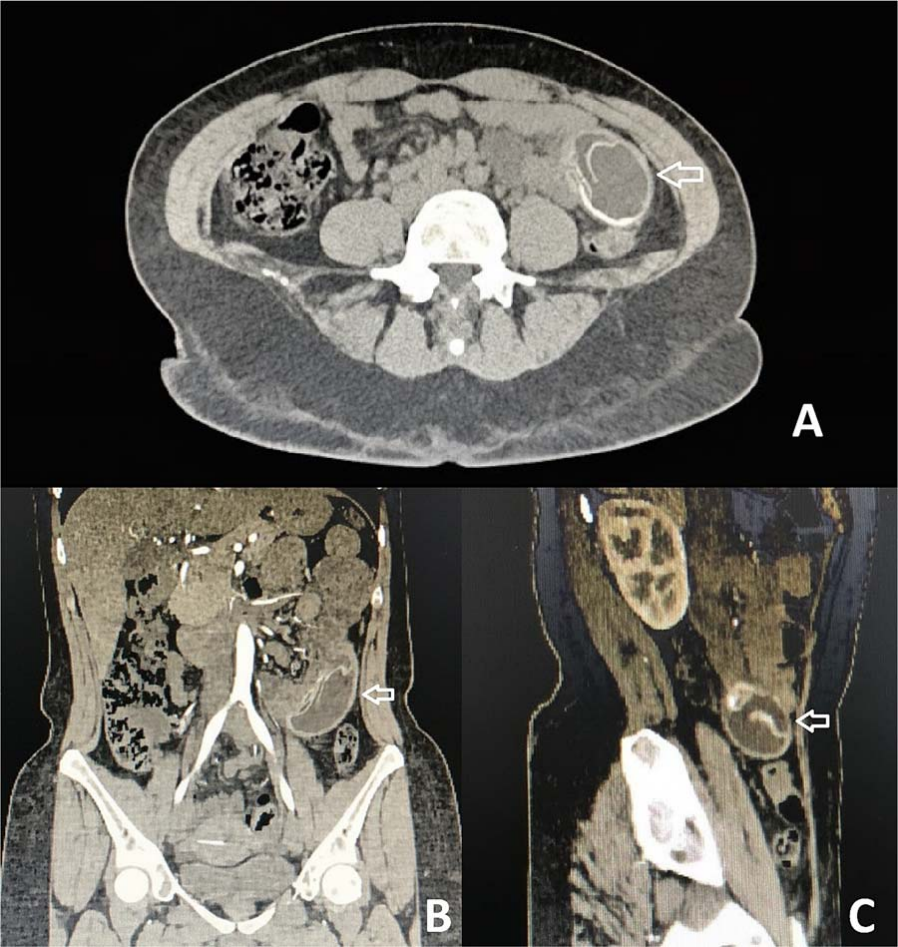
CT with evidence of a foreign body compatible with a migrated gastric balloon (indicated by the arrow). (A) Axial plane. (B) Coronal plane. (C) Sagittal plane.

On examination, her abdomen was mildly distended, tender in the right iliac fossa and hypogastrium, with a positive Blumberg sign. Laboratory testing revealed leukocytosis (10 880/mm^3^) without neutrophilia. Ultrasound findings were unremarkable, but contrast-enhanced computed tomography (CT) demonstrated a foreign body within the jejunum, with proximal small bowel dilatation and a clear transition point—highly suggestive of gastric balloon migration and obstruction (Fig. 1).

**Figure 2:**
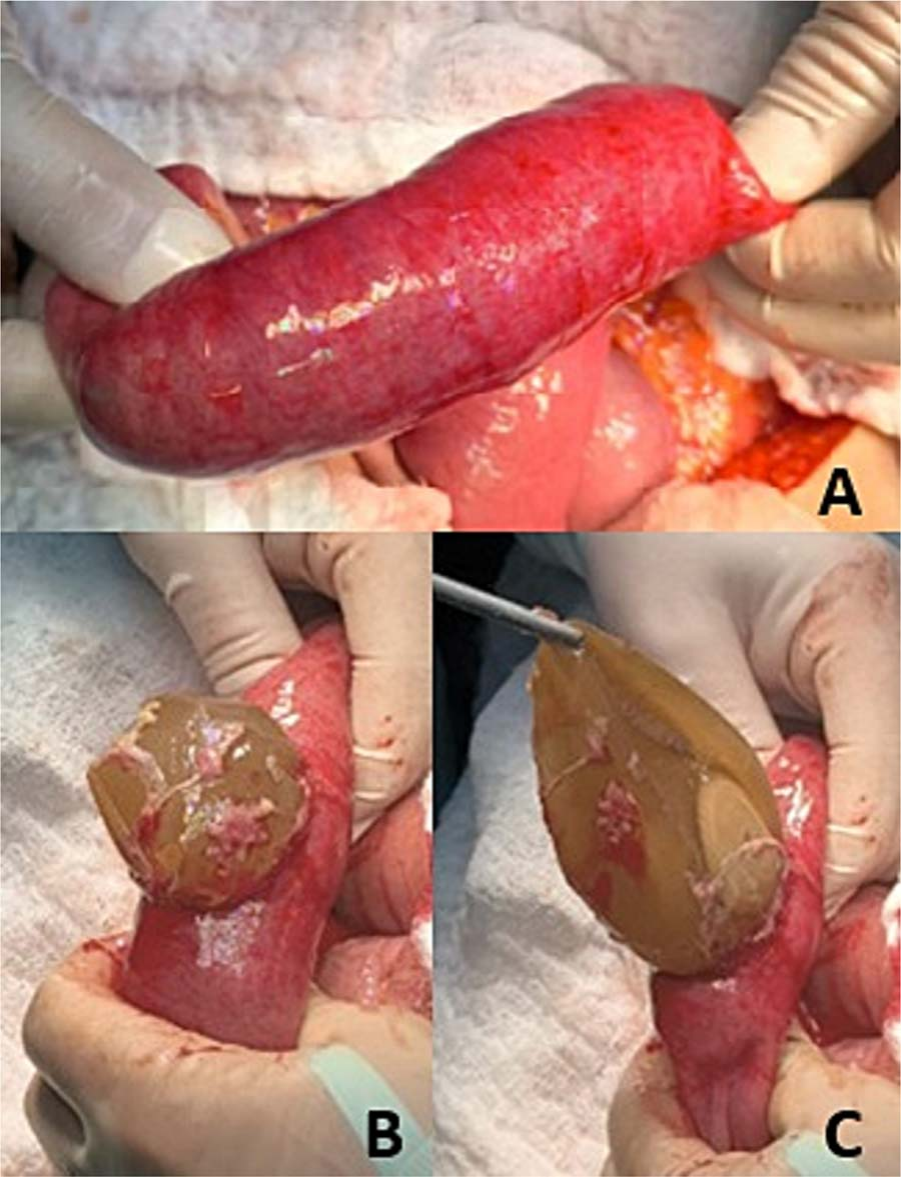
Intestinal obstruction. (A) Identification of the area of intestinal obstruction. (B) Enterectomy. (C) Removal of intraluminal foreign body (migrated gastric balloon).

**Figure 3:**
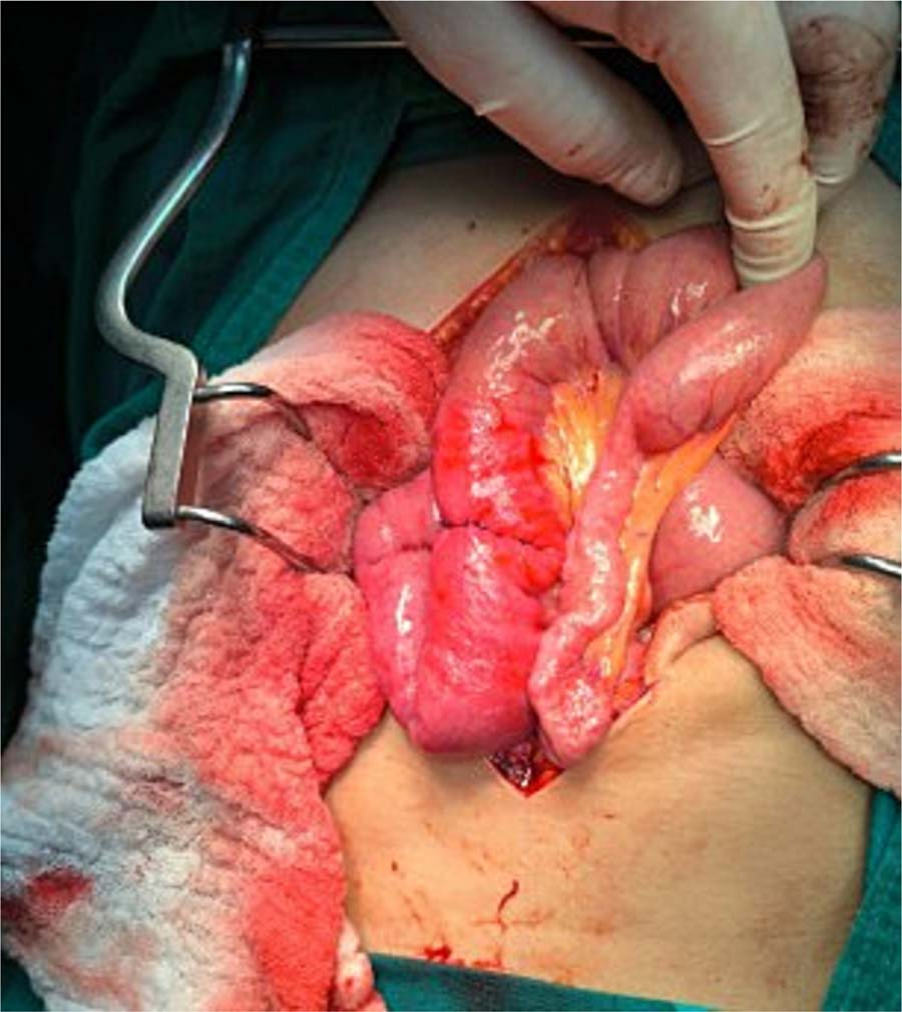
Transverse enterotomy raffia after removal of intraluminal foreign body (migrated gastric balloon).

An exploratory laparotomy confirmed distended stomach and proximal loops, with the balloon impacted ~60 cm distal to the ligament of Treitz, causing complete intraluminal obstruction (Fig. 2A). A transverse enterotomy was performed to extract the foreign body (Fig. 2B and C), followed by primary closure using absorbable polyglycolic suture (Fig. 3). The patient’s postoperative course was uneventful, and she was discharged on the fourth postoperative day.

## Discussion

Although most IGB complications are minor (nausea, vomiting, reflux), migration leading to SBO is a rare but serious event. Migration typically follows balloon deflation, permitting passage through the pylorus into the small intestine [1, 5, 6].

### Comparative analysis of reported cases (Table 1)

**Table 1:** Comparative summary of reported cases of small bowel obstruction secondary to intragastric balloon migration.

**Author/Year**	**Age/Sex**	**BMI/Obesity Grade**	**Time from IGB Insertion to Event**	**Clinical Presentation**	**Diagnostic Modality**	**Location of Balloon**	**Management**	**Surgical/Endoscopic Approach**	**Outcome**
Ntyl *et al*. [5], 2023	17 / F	48.8 (morbid obesity)	Not specified	Early obstruction symptoms (vomiting, pain)	CT abdomen	Small bowel	Laparoscopic removal	Emergency laparoscopy	Full recovery
Bamakhrama *et al*. [6], 2023	33 / F	Not specified	2 months	Epigastric pain, nausea, vomiting	CT abdomen	Proximal jejunum	Endoscopic retrieval	Endoscopic extraction with surgical backup	Full recovery
Di Saverio *et al*. [9], 2021	49 / F	31.2 (obesity grade I	9 months	Cramp-like pain, vomiting, no flatus/stools	X-ray + CT (triple contrast)	Distal jejunum	Laparoscopic enterotomy + intracorporeal suture	Laparoscopic surgery	Full recovery
Brizuela *et al*. [7], 2022	30 / F	Not specified	18 months (delayed removal)	Severe abdominal pain, obstruction symptoms	CT abdomen	Not specified	Surgical enterotomy	Laparotomy	Full recovery
Moszkowicz & Lefevre [8], 2020	49 / M	Not specified	11 months	Acute bowel obstruction	CT abdomen	Ileum	Enterostomy and suture	Laparotomy	Full recovery
Rzepa *et al*. [10], 2019	Adult / M	28 (overweight)	Not specified	Abdominal pain, IGB deflation	CT abdomen	Ileum	Enterotomy	Laparoscopy	Full recovery
Handaya *et al*. [11], 2020	46 / F	Not specified	10 months	Gastric distension, salivation, nausea	CT scan + X-ray	40 cm before ileocecal junction	Ileotomy	Laparoscopy	Full recovery, Discharged after 5 days
Mousavi Naeini & Sheikh [12], 2021	25 / M	Not specified	5 months	Abdominal pain, methylene blue urine	CT + Endoscopy	Not specified	Enterotomy	Surgery	Full recovery
Muñoz-Palomeque *et al*., 2025 (This Case)	37 / F	24.09 (post-obesity)	12 months	Bilious vomiting, abdominal pain, no flatus	CT abdomen	60 cm distal to Treitz	Enterotomy + transverse closure (Vicryl)	Laparotomy	Uneventful recovery, discharged on day 4

#### Clinical presentation

Our patient’s presentation—progressive abdominal pain, bilious vomiting, absence of flatus—parallels the 17-year-old woman reported by Ntyl *et al*. [5], who developed SBO due to balloon migration and was treated laparoscopically with full recovery. Bamakhrama *et al*. [6] described a 33-year-old woman presenting two months post-placement with jejunal obstruction successfully treated by endoscopic retrieval, emphasizing that endoscopic removal may be feasible in partial obstruction without perforation.

#### Timing and risk factors

Most events occur within 6–12 months after insertion, often linked to delayed retrieval or lost follow-up. Brizuela *et al*. [7] reported severe complications 18 months post-placement, highlighting the risk of extended retention. Moszkowicz and Lefevre [8] similarly noted SBO 11 months after insertion of an air-filled balloon. Our case occurred 12 months after placement, again suggesting prolonged retention and unnoticed deflation.

#### Diagnosis

CT scanning remains the diagnostic gold standard, confirming balloon location, obstruction level, and ruling out perforation. Di Saverio *et al*. [9] used triple-contrast CT to identify a distal jejunal obstruction from a BioEnterics balloon before laparoscopic removal. CT accuracy and rapid interpretation allow for early surgical planning, as demonstrated both in their case and ours.

#### Management

Approach depends on obstruction severity, balloon integrity, and migration site:

Endoscopic retrieval—as in Bamakhrama *et al*. [6]—for proximal, partially obstructive, or accessible balloons.

Laparoscopic enterotomy—as performed by Ntyl *et al*. [5], Di Saverio *et al*. [9], and Rzepa *et al*. [10]—for complete obstruction in stable patients.

Open laparotomy—as in our case and Handaya *et al*. [11]—for marked distension, uncertain anatomy, or distal impaction.

All cases reported favorable outcomes when intervention was timely, reinforcing that early recognition and prompt surgery prevent perforation and peritonitis.

#### Proposed clinical guideline for suspected balloon migration

*Clinical suspicion.* Any patient with a history of IGB therapy presenting with abdominal pain, vomiting, or constipation should raise immediate suspicion of migration.

*Initial assessment.*

- Full physical exam for distension, tenderness, rebound.
- Laboratory studies for leukocytosis or metabolic derangements.
- Abdominal X-ray: may show dilated loops but low specificity.

*Imaging confirmation.*

- Contrast-enhanced CT: defines migration site, degree of obstruction, and excludes perforation.
- CT is essential prior to any attempted endoscopic or surgical retrieval.

*Management algorithm.*

- Partial obstruction, proximal location, no perforation: Endoscopic retrieval under surgical standby [6].
- Complete obstruction, distal migration Laparoscopic or open enterotomy [5, 9, 11].
- Perforation, diffuse peritonitis, or uncertain anatomy Urgent open laparotomy

*Postoperative care and prevention.*

- Early mobilization and diet progression as tolerated.
- Documentation of balloon excretion or endoscopic removal within recommended duration (4–6 months).
- Scheduled radiologic follow-up if spontaneous passage is expected (EIGB).

## Conclusions

Despite widespread perception of safety, intragastric balloon therapy is not risk-free. Migration-related SBO remains a rare but significant event that may manifest months after therapy completion. The case described emphasizes the pivotal role of CT diagnosis, timely surgical intervention, and long-term follow-up.

Clinicians should maintain vigilance for persistent or late abdominal symptoms in post-IGB patients and adopt structured surveillance to confirm balloon retrieval or passage. Awareness of this potential complication ensures safe continuation of a valuable minimally invasive therapy for obesity.
